# Interactions Between Socioeconomic Status and Mental Health Outcomes in the Nigerian Context Amid COVID-19 Pandemic: A Comparative Study

**DOI:** 10.3389/fpsyg.2020.559819

**Published:** 2020-10-06

**Authors:** Samson F. Agberotimi, Olusola S. Akinsola, Rotimi Oguntayo, Abayomi O. Olaseni

**Affiliations:** ^1^Lifestyle Diseases Research Entity, North-West University, Mafikeng, South Africa; ^2^Department of Psychology, University of Ibadan, Ibadan, Nigeria; ^3^Department of Psychology, University of Ilorin, Ilorin, Nigeria

**Keywords:** COVID-19, mental health, socioeconomic status, healthcare workers, Nigeria

## Abstract

This study examines the mental health outcomes among the healthcare personnel and the general population and the role of socioeconomic status. Eight hundred and eighty-four (884) residents in Nigeria comprising 382 healthcare personnel and 502 general residents aged between 18 to 78 years (M = 28.75, SD = 8.17) responded to an online survey with measures of Impact of Event Scale-Revised (IES-R), Generalized Anxiety Disorder (GAD-7), Patient Health Questionnaire (PHQ–9), and Insomnia Severity Index. Collected data were subjected to statistical analysis using the SPSS v.25. Results revealed significant difference in the prevalence of depressive symptoms (χ^2^ = 14.26; df = 4; *p* < 0.01), insomnia symptoms (χ^2^ = 40.21; df = 3; *p* < 0.01), posttraumatic stress symptoms (χ^2^ = 08.34; df = 3; *p* < 0.05), and clinical anxiety symptoms (χ^2^ = 06.71; df = 1; *p* < 0.05) among healthcare personnel and the general population, with a higher prevalence reported by the healthcare personnel. Further, socioeconomic status significantly influences prevalence of depressive symptoms (χ^2^ = 04.5; df = 4; *p* < 0.05). The study concluded that the prevalence of poor mental health outcomes during the COVID-19 crisis among Nigerians is worrisome. Also, the socioeconomic status of the citizens has serious implications on depressive symptoms. The study recommends that the government and stakeholders should pay attention to policy that will favor tele-mental health services and adequate palliative measures to cushion the psycho-economic impacts of COVID-19 on residents. Also, healthcare workers should be considered for better remuneration and other welfare benefits to sustain their well-being during the present and future pandemic.

## Introduction

Since the outbreak of coronavirus, otherwise known as COVID-19, which was first reported in December 2019 in Wuhan China, declared as a Public Health Emergency of International Concern in January 2020 and later a pandemic in March 2020 by the World Health Organization (WHO), the world has not been the same (World Health Organisation, 2020). For instance, the statistics released on August 28, 2020, indicated that there had been more than 24.6 million confirmed cases of COVID-19 and 835,000 deaths worldwide (Worldometer, 2020). Worldometer reported further that, at the end of August 2020 in Nigeria, more than 53,317 cases have been confirmed, while over 1,011 people have died (Worldometer, 2020). Despite the disturbing figures, it has been opined that the actual global incidence rate of COVID-19 cases is likely to be far higher than what the statistics show (Flaxman et al., 2020).

Being a novel disease that is highly contagious, spreading fast across the world, and the fact that there is yet to be an established cure for it, the COVID-19 pandemic has created a lot of panic in every part of the world. In response, many countries have put up different measures, especially those upholding social distancing order, to slow down the spread of the disease. Although there is evidence of the effectiveness of quarantine measures to control the spread of infectious diseases such as cholera, severe acute respiratory syndrome (SARS), or Ebola in the past (e.g., Twu et al., 2003), the effect of restriction of movement and lockdown on socioeconomic activities across different countries of the world has generated different opinions about the impact on the general citizens (Goldman et al., 2018; Forbes and Krueger, 2019; Mbamalu, 2019; The World Bank, 2020).

From the foregoing, emerging evidence has implicated the ongoing COVID-19 pandemic in the mental health outcomes among different populations such as the healthcare professional and the general public worldwide (e.g., Ji et al., 2017; Brooks et al., 2020; Lai et al., 2020; Olaseni et al., 2020; and Rossi et al., 2020). Besides, the social-distancing and self-isolation during the COVID-19 pandemic place more challenges on the mental health and general well-being of the people (Mukhtar, 2020). In addition, it is crucial to assess the mental health outcomes of people during a global crisis such as the COVID-19 pandemic alongside the socioeconomic status and attributes of the people. This is because several studies have established strong relationships between socioeconomic status (SES) and mental well-being. More importantly, low SES have been implicated in poor self-reported mental health including depression, anxiety, sleep problems, and psychological distress in adults and adolescents (Richter et al., 2009; Salami and Walker, 2014; Pappas, 2020).

Having a holistic understanding of the mental health outcomes of all members of the society, not only focusing on direct victims (patients) and healthcare providers in response to any major event, is crucial to the recovery of the people. This position was emphasized by the proposition of the Canadian National Advisory Committee on SARS and Public Health in 2003 that a “systemic perspective,” focusing on the general population and not just medical staff and patients should be embraced in addressing the SARS epidemic (Naylor et al., 2003). Nevertheless, the literature shows that many studies addressing the psychological consequences of COVID-19 are focusing exclusively on either the healthcare professionals or the general public, therefore limiting the opportunity of comparison between the two populations concerning the mental health outcomes during the COVID-19 crisis.

Furthermore, the situation in Nigeria calls for special attention because being a developing country where small medium enterprises (SMEs) contributed 48% of the national gross domestic products (GDP) that account for 96% of the businesses and 84% of employment (Public Works Corporation, 2020), the effect of the present COVID-19 pandemic and lockdown on socio-economic activities is likely to be severe on the well-being of the people, many of whom rely on daily income to cater for their personal and family financial needs. Hence, the significance of this study addresses that the interaction between socioeconomic status and mental health outcomes of Nigerians during the COVID-19 pandemic cannot be overemphasized. Specifically, the following objectives guided this study:

1. To investigate the interaction between socioeconomic status of respondents and mental health outcomes during the COVID-19 crisis.
2. To compare the mental health outcomes of healthcare workers with the general public during the COVID-19 pandemic.

## Materials and Methods

### Design

This research was a web-based cross-sectional survey that was conducted via social media (Facebook and WhatsApp posts) using a Google form from March 20 to April 19, 2020. This was found appropriate to enable the investigators to assess the psychological distress experienced by participants during the COVID-19 pandemic without manipulating the variables of interest.

### Sampling

A snowball sampling technique was utilized in this study. This method was considered appropriate due to the imposed restrictions of movement and lockdown in Nigeria during the period. Key persons (e.g., known healthcare workers or frontline staff, friends, colleagues, and those on the contact of the researchers) across each category of respondents (healthcare workers and the general population) were considered the seed in the study, and they were encouraged to disseminate the links with others in the same category accordingly. The online semi-structured questionnaire developed using Google forms with an appended consent form was sent through emails, WhatsApp, and Facebook platforms to potential respondents on the contact of the investigators. Those prospective individuals were then encouraged to roll out the survey to other colleagues or residents in Nigeria.

### Participants

Eight hundred and eighty-four (884) participants were involved in the current study. The sampled respondents cut across healthcare personnel and the general public. Healthcare personnel were 43.21% (*n* = 382), while the general public constituted 56.79% (*n* = 502) of the study respondents. Considering the gender disparity, the majority of the respondents were male, which constituted the 54.5% of the total respondents, while the female counterparts constituted 45.5%. There was a disparity in the distribution of geo-political zones of the respondents; the majority of the respondents were from the southern part of Nigeria, constituting 85.2% of the sample, 10.9% were from the northern part of Nigeria, while 4% were foreign residents in Nigeria. The disparity across respondents’ marital status and level of education were also reported (see Table 1).

**TABLE 1:** Socio-demographic characteristics of the study participants.

Characteristics	Overall (*N* = 884/%)	Healthcare personnel (*n* = 382/%)	General population (*n* = 502/%)
**Sex**			
Male	482 (54.5)	207 (54.2)	269 (53.6)
Female	402 (45.5)	169 (44.2)	225 (44.8)
**Religion affiliation**			
Christianity	609 (68.9)	272 (71.2)	337 (67.1)
Islam	261 (29.5)	102 (26.7)	159 (31.7)
Others	14 (01.6)	08 (2.1)	06 (1.2)
**Regional affiliation**			
Southern, Nigeria	753 (85.2)	319 (83.5)	433 (86.3)
Northern, Nigeria	96 (10.9)	44 (11.5)	47 (9.4)
Foreigner	35 (4.0)	17 (04.4)	22 (4.4)
**Marital status**			
Single	297 (33.6)	132 (34.6)	165 (32.9)
Married	577 (65.3)	244 (63.9)	333 (66.3)
Separated/divorced	10 (01.1)	06 (01.6)	04 (0.8)
**Level of Education**			
Bachelor degree and its equivalent	352 (39.8)	154 (40.3)	198 (39.4)
Diploma and its equivalent	236 (26.7)	100 (26.2)	136 (27.1)
Postgraduate education	254 (28.7)	108 (28.3)	146 (29.1)
Secondary school education	42 (04.8)	20 (05.2)	22 (04.4)

### Instrument

Data were collected via an online self-reported questionnaire designed by the investigators. The questionnaire contained six sections related to the mental health outcome of health workers in Nigeria amid the coronavirus pandemic. The first section consisted of information assessing demographic attributes such as sex, age, religion, and marital status of participants.

The second section contained the 22-item of the Impact of Event Scale-Revised (IES-R) (Weiss, 2007). The scale was developed to measure the subjective response of an individual to a specific traumatic event, especially the response to sets of intrusion, avoidance, and hyperarousal, as well as total subjective stress. The IES-R is not a diagnostic tool but just a screening measure. The total IES-R score was divided into 0–23 (normal), 24–32 (mild psychological impact), 33–36 (moderate psychological impact), and >37 (severe psychological impact). Briere (1997) affirmed the validity and reliability of the scale. Cronbach’s alpha 0.82 was established as the reliability coefficient for the scale in this study.

Section three of the questionnaire was the GAD-7 (Spitzer et al., 2006). It consisted of 7 questions assessing generalized anxiety disorder. The items focused on the frequency of symptoms during the preceding 2-week period of COVID-19 lockdown in Nigeria. The GAD-7 requires approximately 1–2 min to administer and for each symptom queried provides the following response options: “not at all,” “several days,” “over half the days,” and “nearly every day” and these items are scored as 0, 1, 2, or 3, respectively. A score ranging from 0 to 21 is obtainable by respondents. Scores of 5, 10, and 15 are taken as the cut-off points for mild, moderate, and severe anxiety, respectively. Cronbach’s alpha 0.81 was established as the reliability coefficient for the scale in this study.

The fourth section contained the Patient Health Questionnaire (PHQ–9). The PHQ–9 is a nine-item depression scale that has the potential of performing a dual-purpose of the instrument. It can establish the diagnosis of a depressive disorder and reveal the grade of symptom severity (Kroenke et al., 2001). Statements measuring depressive symptoms such as “little interest/pleasure in doing things” were rated from 0 (not at all) to 3 (nearly every day) by respondents as applicable to them over the past two weeks during the lockdown. PHQ-9 scores can range from 0 to 27. The scale has strong psychometric properties (e.g., Botha, 2011) and has been widely used. Cronbach’s alpha 0.87 was established as the reliability coefficient for the scale in this study.

The fifth section contained the Insomnia Severity Index; this is a 7-item self-report questionnaire assessing the nature, severity, and impact of insomnia. Participants were required to rate their sleep condition in the last 2 weeks as described by each item of the scale. Questions on the ISI cut across the severity of sleep onset, sleep maintenance, and early morning awakening problems, sleep dissatisfaction, interference of sleep difficulties with daytime functioning, noticeability of sleep problems by others, and distress caused by the sleep difficulties. The scale is responded to on a 5-point Likert scale with a score ranging from 0 to 4, thus yielding a total score ranging from 0 to 28. The total score is interpreted as follows: the absence of insomnia (0–7); sub-threshold insomnia (8–14); moderate insomnia (15–21); and severe insomnia (22–28). Previous studies have reported adequate psychometric properties for both the English and French versions (e.g., Bastien et al., 2001). Cronbach’s alpha 0.78 was established as the reliability coefficient for the scale in this study.

### Procedure

The study was an online cross-sectional survey study. Only adults (aged between 18 and 78 years) who were either healthcare workers or Nigerian residents with access to the internet were involved in this study. Also, participants must be able to read and understand in basic English language and be willing to click the agree button to participate before having to access the survey. A link to the survey on Google form was sent to all participants. On receiving and clicking the link, the participants got auto-re-directed to the survey items. A detailed informed consent form was attached at the beginning of the online questionnaire and only individuals who gave their consent participated in the study. The data collection was initiated on March 20, 2020, and closed on April 19, 2020. The sampling technique utilized allowed the investigators to collect data from across various states of Nigeria. Eight hundred and eighty-four (884) correctly filled questionnaires were recovered through the Google form and processed for statistical analyses.

### Data Analysis

The collected data were analyzed using the Statistics Package of Social Sciences (SPSS; version 25). The analyzed data responded to the two research questions stated earlier in this study. The analyses included reliability coefficients of the used scales, prevalence estimate analysis, and chi-square analysis.

## Results

This section presents the results and interpretation of the data collected. The analyses of the interaction between socioeconomic status and mental health outcomes of selected respondents (healthcare personnel and the general population) in Nigeria during the COVID-19 pandemic lockdown were conducted. The results captured the socio-demographic characteristics of the respondents as well as the interaction between socioeconomic status and mental health outcomes of respondents which are presented in Tables 1–3.

Outcomes of the study (see Table 2) revealed that there was a significant difference in the prevalence of depressive symptoms among respondents with different socioeconomic status (χ^2^ = 04.05; df = 4; *p* < 0.05). Furthermore, it was found that the prevalence of clinical depressive symptoms was significantly higher among respondents with the standard income compare to those above standards and below standard incomes (61.5% vs. 22.8% and 20.2%; 95% CI, 0.63–4.60; *p* < 0.05). Further findings revealed that there was no significant difference in the prevalence of insomnia symptoms among categories of socioeconomic status (χ^2^ = 02.38; df = 3; *p* > 0.05). However, the prevalence of clinical insomnia symptoms was insignificantly higher among respondents with standard income compared to respondents with the above standard and below standard incomes (60.1% vs. 24.9% and 15.3%; 95% CI, 0.20–1.68; *p* > 0.05).

**TABLE 2:** Showing the interaction between socioeconomic status of respondents and the prevalence of depression, insomnia, and posttraumatic stress symptoms and anxiety in Nigeria (*N* = 884).

Outcome	Above standard income (*n* = 201*%)	Standard income (*n* = 516*%)	Below standard income (*n* = 167*%)	Prevalence ratio (95% CI)	χ^2^	*p*
Depression	46 (22.8)	155 (61.5)	51 (20.2)	0.63 – 4.60	04.05	<0.05
Insomnia	50 (24.9)	122 (60.1)	31 (15.3)	0.20 – 1.68	02.38	>0.01
PTSS	87 (43.3)	249 (48.3)	80 (47.9)	0.53 – 2.98	01.50	>0.05
Anxiety	91 (45.3)	242 (46.9)	79 (47.3)	0.49 – 2.83	00.19	>0.05

*Note: Clinical severity in the study outcomes are the reference categories of all variables.*

Similarly, study findings revealed that there was no significant difference in the prevalence of posttraumatic stress symptoms (PTSS) among different socioeconomic classes (χ^2^ = 01.50; df = 3; *p* > 0.05). However, the prevalence of posttraumatic symptoms was insignificantly higher among respondents with standard income compared to respondents with the above standard and below standard incomes (48.3% vs. 43.3% and 47.9%; 95% CI, 0.53–2.98; *p* > 0.05). Insignificant difference was also reported in the prevalence of clinical anxiety symptoms among classes of socioeconomic status (χ^2^ = 0.19; df = 1; *p* > 0.05). However, the prevalence of anxiety symptoms was insignificantly higher among respondents with standard income compared to respondents with the above standard and below standard incomes (46.9% vs. 45.3% and 47.3; 95% CI, 0.49–2.83; *p* > 0.05).

Another objective of the study proposed to examine the comparative analysis of mental health outcomes among the healthcare population and the general population was presented in Table 3.

**TABLE 3:** Prevalence of depression, insomnia, and posttraumatic stress symptoms and anxiety in healthcare personnel and general population (*N* = 884).

Outcome	Healthcare personnel (*n* = 382*%)	General population (*n* = 502*%)	Adjusted prevalence ratio (95% CI)	χ^2^	*p*
Depression	134 (35.1)	118 (23.5)	0.537 – 1.034	14.26	<0.01
Insomnia	127 (33.2)	76 (15.1)	0.289 – 0.579	40.21	<0.01
PTSS	201 (52.6)	215 (42.8)	0.847 – 1.519	08.34	<0.05
Anxiety	223 (58.4)	249 (49.6)	0.719 – 1.307	06.71	<0.05

*Note: Clinical severity in the study outcomes are the reference categories of all variables.*

Responses from 884 participants in the study (i.e., both healthcare personal and the general population) were screened for depression, anxiety, insomnia, and posttraumatic symptoms constituted the outcome of the analysis. Outcomes of the study (see Table 3) revealed that there was a significant difference in the prevalence of depressive symptoms among healthcare personal and the general population (χ^2^ = 14.26; df = 4; *p* < 0.01). Further, it was found that the prevalence of clinical depressive symptoms was significantly higher among healthcare personnel than the general population (35.1% vs. 23.5%; 95% CI, 0.54–1.03; *p* < 0.01). Finding further revealed that there was a significant difference in the prevalence of insomnia symptoms among healthcare personal and the general population (χ^2^ = 40.21; df = 3; *p* < 0.01). Such that, the prevalence of clinical insomnia symptoms was significantly higher among healthcare personnel than the general population (33.2% vs. 15.1%; 95% CI, 0.29–0.58; *p* < 0.01).

Similarly, study findings revealed that there was a significant difference in the prevalence of PTSS among healthcare personal and the general population (χ^2^ = 08.34; df = 3; *p* < 0.05). Such that, the prevalence of posttraumatic symptoms was significantly higher among healthcare personnel than the general population (52.6% vs. 42.8%; 95% CI, 0.85–1.52; *p* < 0.05). A significant difference was also reported in the prevalence of clinical anxiety symptoms among healthcare personal and the general population (χ^2^ = 06.71; df = 1; *p* < 0.05). Such that, the prevalence of anxiety symptoms was significantly higher among healthcare personnel than the general population (58.4% vs. 49.6%; 95% CI, 0.72–1.31; *p* < 0.05).

## Discussion

The objectives of this study are to investigate the interaction between socioeconomic status and mental health outcomes of respondents during the COVID-19 pandemic and to make a comparison between the mental health outcomes of healthcare workers and the general public in Nigeria during the COVID-19 pandemic. Consequently, our study revealed significant differences among the three socioeconomic status classes on depression only, invariably, this means that no significant difference was found among the three socioeconomic status classes on insomnia, posttraumatic stress symptoms and anxiety in Nigeria during the COVID-19. In detail, our study revealed that the standard income socioeconomic class reported almost two-thirds (62%) higher prevalence of depression as against the above standard income and below standard income class (22.8% vs. 20.2%).

Consistent with our findings, various studies have reported the relationship between socioeconomic status and mental health during COVID-19, but there has been a disparity in the class that is most affected. While our study reported the standard (middle) income socioeconomic class having the highest prevalence of depression during the COVID-19 pandemic, Heath (2020) reported 45% of the above standard (upper) income socioeconomic status class reported their emotional well-being harmed by coronavirus as against 34% and 36% from the lower and middle socioeconomic status class. Besides, contrary to our findings, Pappas (2020) suggested that people with lower socioeconomic status have a higher tendency to have mental health issues; however, the low socioeconomic level has been associated with death and high illness rates in several studies, regardless of the cause of death being from infectious or non-infectious diseases and indices for measuring socioeconomic status (Kaplan et al., 2007; Oguntayo et al., 2018).

The reason for our result could be associated to the fact that the standard (middle) income socioeconomic class in Nigeria is the largest socioeconomic group, occupying the wide inequality gap between the haves and the haves not. These individuals are mostly business owners (SMEs) and major salary earners from the private and government establishments; therefore, they are the most hit by the closure of businesses and lockdown in the country. Besides, there is speculation of a looming recession due to the COVID-19 pandemic (The World Bank, 2020), which has spurred some organizations to lay off staff and reduce the salary of the retained staff while so many have not received any salary since the lockdown in Nigeria. In this respect, Holmes et al. (2020) argued that serious psychological distress is anticipated from potential global economic crisis following the COVID-19 pandemic. This argument is substantiated by previous evidence linking the socioeconomic status of the people to their mental health outcome following the SARS epidemic in 2003 (e.g., Nickell et al., 2004; Tsang et al., 2004; Yip et al., 2010; Kanter and Manbeck, 2020). These are valid reasons why this group may have reported a higher prevalence of depression compared to the above standard (high) income class who are mostly politicians and big industrialists that have no fear of layoff or reduction in salary and below standard income class.

The unbalanced prevalence of depression among the socioeconomic classes in our study (22.8% vs. 61.5% vs. 20.2%) confirmed the assertion of Kanter and Manbeck (2020) that a large population may develop inequitably distributed depression due to the stressors of the COVID-19 crisis. Fear of inability to feed, pay house rent, and purchase of basic safety materials such as sanitizer and mandatory nose mask to fit into the new norm have added to the economic burden of the standard income and below standard income class, thereby leading to the prevalence of higher depressive states compared against the above standard income class. Importantly, the above standard income class in Nigeria holds the economic power of the country and economic power translates into political power, thereby giving control of state structure into the hands of the above standard income class. Moreover, various studies have confirmed that mental health deteriorates in line with the level of socioeconomic status (Goldman et al., 2018) and economic recessions (Forbes and Krueger, 2019).

Our study further revealed differences in the mental health outcomes between the Nigerian healthcare workers and the general population during the COVID-19 pandemic. In specifics, the prevalence of depression, insomnia, posttraumatic stress symptoms, and anxiety was higher among the healthcare workers (35.1%, 33.2%, 52.6%, and 58.4%, respectively) as against the prevalence of depression, insomnia, posttraumatic stress symptoms, and anxiety of the general population (23.5%, 15.1%, 42.8%, and 49.6%, respectively). The higher prevalence of mental health outcomes among healthcare workers during the COVID-19 pandemic can hereby be connected with their role as caregivers and essential workers during the pandemic. Interestingly, a similar higher prevalence of mental health outcomes was reported among healthcare workers in China caring for COVID-19 patients as against lower prevalence of mental health outcomes among the Chinese general population (Lai et al., 2020; Wang et al., 2020).

Healthcare workers are essential workers that have to care for the infected despite the impending risk during a pandemic, due to the duty and obligation to care. Compared against the general population that has restricted movement and discontinued working due to the COVID-19 pandemic, healthcare workers are required to be at work, despite the human-to-human transmissible nature of the virus, wear uncomfortable personal protective equipment, work overtime, and observe directly, the devastation of the virus on their patients. These experiences have a more social, emotional, and psychological effect on healthcare workers than the general population as revealed in this study.

## Conclusion

This study exposed the prevalence of mental health outcomes among Nigerians during the COVID-19 pandemic. Specifically, the result revealed a disproportionate prevalence of depression among the three socioeconomic classes, whereby the standard income (middle) socioeconomic class recorded the highest prevalence of depression. Besides, the prevalence of mental health outcomes (depression, insomnia, posttraumatic stress symptoms, and anxiety) was higher among the Nigerian health care workers when compared against the general population in Nigeria.

## Limitation of the Study

This study established the association between socioeconomic status and stress-related behaviors; however, there are still some probable limitations. The participants were relatively few; therefore, this result should be carefully generalized as there is a possibility that outcomes would vary if measured on more numbers of participants. Also, response biases which most times are difficult to eliminate in a self-report survey study like this might have affected respondents’ opinions, thereby limiting the results of this study. All these shortcomings might have influenced this result and limit the external validity of this study.

## Recommendations

Looking at the results of the current study, these recommendations are suggested to alleviate the mental health challenges among the residents and health workers during the pandemic period:

1. Federal and state governments in Nigeria should initiate a bill honoring the tele-mental health services to manage present and future pandemic psychological implications. This will help to integrate psychological and medical health services in the fighting against any disease outbreak in the country, especially when face-to-face appointments are risky.
2. Healthcare stakeholders needed to collaborate with psychotherapists in the management of pandemic or disease outbreak to regulate residents’ emotions and that of self to promote wholistic well-being in Nigeria.
3. Stakeholders in government should pay attention to policy that will favor adequate palliative measures to cushion the economic impacts of COVID-19 on the mental health of residents in Nigeria. Also, healthcare workers should be considered for better remuneration and other economic benefits to sustain their well-being during the present and future pandemic.

## Data Availability Statement

The datasets presented in this study can be found in online repositories. The names of the repository/repositories and accession number(s) can be found in the article/Supplementary Material.

## Ethics Statement

Ethical review and approval was not required for the study on human participants in accordance with the local legislation and institutional requirements. Written informed consent was implied via completion of the questionnaire/survey.

## Author Contributions

RO and SA contributed to the conception and design of the study. SA, OA, RO, and AO contributed to the acquisition of data. AO contributed to the analysis and interpretation of data. RO, OA, and SA contributed to the drafting of the manuscript. OA contributed to the critical revision of the manuscript. All authors read and approved the final manuscript for publication.

## Conflict of Interest

The authors declare that the research was conducted in the absence of any commercial or financial relationships that could be construed as a potential conflict of interest.

## References

[B1] Bastien C. H. Vallieres A. Morin C. M. (2001). Validation of the insomnia severity index as an outcome measure for insomnia research. *Sleep Med.* 2 297–307. 10.1016/s1389-9457(00)00065-4 11438246

[B2] Botha M. N. (2011). *Validation of the Patient Health Questionnaire (PHQ-9) in an African Context.* Available online at: http://hdl.handle.net/10394/4647 (accessed May 3, 2020).

[B3] Briere J. (1997). *Psychological Assessment of Adult Posttraumatic States*. Washington D.C.: American Psychological Association.

[B4] Brooks S. K. Webster R. K. Smith L. E. Woodland L. Wessely S. Greenberg N. (2020). The psychological impact of quarantine, and how to reduce it: rapid review of the evidence. *Electron. J.* 395 912–920. 10.1016/s0140-6736(20)30460-8 PMC7158942 32112714

[B5] Flaxman S. Mishra S. Gandy A. Unwin H. Coupland H. Mellan T. (2020). Estimating the number of infections and the impact of non-pharmaceutical interventions on COVID-19 in 11 European countries. *Nature* 584 257–261. 10.25561/77731 32512579

[B6] Forbes M. K. Krueger R. F. (2019). The great recession and mental health in the United States. *SAGE J.* 7 900–913. 10.1177/2167702619859337 32775044 PMC7413622

[B7] Goldman N. Glei D. A. Weinstein M. (2018). Declining mental health among disadvantaged Americans. *Proc. Natl. Acad. Sci. U.S.A.* 115 7290–7295. 10.1073/pnas.1722023115 29915079 PMC6048554

[B8] Heath S. (2020). *Socioeconomic Status Tied to Mental Health During Coronavirus. Patient Engagement Hit.* Available online at: https://patientengagementhit.com/news/socioeconomic-status-tied-to-mental-health-during-coronavirus (accessed March 23, 2020).

[B9] Holmes E. A. O’Connor R. C. Perry V. H. Tracey I. Wessely S. Arseneault L. (2020). Multidisciplinary research priorities for the COVID-19 pandemic: a call for action for mental health science. *Lancet Psychiatry* 7 547–560. 32304649 10.1016/S2215-0366(20)30168-1 PMC7159850

[B10] Ji D. Ji Y. Duan X. Li W. Sun Z. Song X. (2017). Prevalence of psychological symptoms among Ebola survivors and healthcare workers during the 2014-2015 Ebola outbreak in Sierra Leone: a cross-sectional study. *Oncotarget* 8 12784–12791. 10.18632/oncotarget.14498 28061463 PMC5355054

[B11] Kanter J. Manbeck K. (2020). *COVID-19 Could Lead to an Epidemic of Clinical Depression and the Health System isn’t Ready for (that), Either. The Conversation.* Available online at: https://theconversation.com/covid-19-could-lead-to-an-epidemic-of-clinical-depression-and-the-health-care-system-isnt-ready-for-that-either-134528 (accessed March 23, 2020).

[B12] Kaplan G. A. Haan M. N. Syme L. Minker M. Winkleby M. (2007). Socioeconomic status and health. *Clos. Gap* 125–129.

[B13] Kroenke K. Spitzer R. L. Williams J. B. (2001). The PHQ-9: validity of a brief depression severity measure. *J. Gen. Intern. Med.* 16 606–613. 10.1046/j.1525-1497.2001.016009606.x 11556941 PMC1495268

[B14] Lai J. Ma S. Wang Y. Cai Z. Hu J. Wei N. W. (2020). Factors associated with mental health outcomes among healthcare workers exposed to coronavirus disease 2019. *JAMA Netw. Open* 3:976. 10.1001/jamanetworkopen.2020.3976 32202646 PMC7090843

[B15] Mbamalu S. (2019). *Nigeria has a Mental Problem.* London: Aljazeera.

[B16] Mukhtar S. (2020). Pakistanis’ Mental Health during the COVID-19. *Asian J. Psych.* 51 102127. 10.1016/j.ajp.2020.102127 32344330 PMC7179483

[B17] Naylor D. Basrur S. Bergeron M. G. Brunham R. C. Butler-Jones D. Dafoe G. (2003). *Learning from SARS: Renewal of Public Health in CANADA.* Ottowa: National Advisory Committee on SARS and Public Health.

[B18] Nickell L. A. Crighton EJ Tracy CS Al-Enazy H Bolaji Y Hanjrah S. (2004). Psychosocial effects of SARS on hospital staff: survey of a large tertiary care institution. *Can. Med. Assoc. J.* 170 793–798. 10.1503/cmaj.1031077 14993174 PMC343853

[B19] Oguntayo R. Oyeleke J. T. Popoola O. A. Opayemi A. S. Faworaja O. R. (2018). Influence of socio-economic factors on domestic violence among couples in Ibadan metropolis. *ESUT J. Psychol. Sci.* 3 14–25.

[B20] Olaseni A. O. Akinsola O. S. Agberotimi S. F. Oguntayo R. (2020). Psychological distress experiences of nigerians amid COVID-19 pandemic. *Social Sciences & Humanities Open* 2 2590–2911. 10.1016/j.ssaho.2020.100052 PMC7448734 34173493

[B21] Pappas S. (2020). *How Will People React to the New Financial Crisis?.* Washington, DC: American Psychological Association.

[B22] Public Works Corporation (2020). *Assessing Current Market Conditions and Business Growth Prospects.* Available online at: https://www.pwc.com/ng/en/events/nigeria-sme-survey.html (accessed March 23, 2020).

[B23] Richter M. Erhart M. Vereecken C. A. Zambon A. Boyce W. Gabhainn S. N. (2009). The role of behavioural factors in explaining socio-economic differences in adolescent health: a multilevel study in 33 countries. *Soc. Sci. Med.* 69 396–403. 10.1016/j.socscimed.2009.05.023 19540029

[B24] Rossi R. Socci V. Talevi D. Mensi S. Niolu C. Pacitti F (2020). COVID-19 pandemic and lockdown measured impact on mental health among the general population in Italy. *Front. Psychiatry* 11:790. 10.3389/fpsyt.2020.00790 32848952 PMC7426501

[B25] Salami T. K. Walker R. L. (2014). Socioeconomic status and symptoms of depression and anxiety in African American college students: the mediating role of hopelessness. *J. Black Psychol.* 40 275–290. 10.1177/0095798413486158

[B26] Spitzer R. L. Kroenke K. Williams J. B. Löwe B. (2006). A brief measure for assessing generalized anxiety disorder: the GAD-7. *Arch. Intern. Med.* 166 1092–1097. 10.1001/archinte.166.10.1092 16717171

[B27] The World Bank (2020). *COVID-19 (Coronavirus) Drives Sub-Saharan Africa Toward First Recession in 25 Years.* Washington, DC: The World Bank.

[B28] Tsang H. W. Scudds R. J. Chan E. Y. (2004). Psychosocial impact of SARS. *Emerg. Infect. Dis.* 10 1326–1327. 10.3201/eid1007.040090 15338536 PMC3323309

[B29] Twu S.-J. Chen T.-J. Chen C.-J. Olsen S. J. Lee L.-T. Fisk T. (2003). Control measures for severe acute respiratory syndrome (SARS) in Taiwan. *Emerg. Infect. Dis.* 9 718–720. 10.3201/eid0906.030283 12781013 PMC3000163

[B30] Wang C. Pan R. Wan X. Tan Y. Xu L. Ho C. S. (2020). Immediate psychological responses and associated factors during the initial stage of the 2019 Coronavirus Disease (COVID-19) epidemic among the general population in China. *Intern. J. Environ. Res. Public Health* 17:1729. 10.3390/ijerph17051729 32155789 PMC7084952

[B31] Weiss D. S. (2007). “The impact of event scale: revised,” in *Cross-Cultural Assessment of Psychological Trauma and PTSD*, eds Wilson J. P. Tang C. S. (New York: Springer), 219–238.

[B32] World Health Organisation (2020). *Advice and Guidance from WHO on COVID-19.* Available online at: https://www.who.int/emergencies/diseases/novel-coronavirus-2019 (accessed March 15, 2020).

[B33] Worldometer (2020). *COVID-19 Coronavirus Pandemic.* Available online at: https://www.worldometers.info/coronavirus/ (accessed August 15, 2020).

[B34] Yip P. S. Cheung Y. T. Chau P. H. Law Y. W. (2010). The impact of epidemic outbreak: the case of severe acute respiratory syndrome (SARS) and suicide among older adults in Hong Kong. *Crisis* 31 86–92. 10.1027/0227-5910/a000015 20418214

